# Concomitant Mineral Carbonation and CO_2_ Reduction in Short‐Term Water‐Basalts Reaction: Difference Between Basaltic Glass and Basaltic Crystals

**DOI:** 10.1002/gch2.70147

**Published:** 2026-08-12

**Authors:** Andrea Pierozzi, Rémi Rateau, Andrea Orlando, Daniele Borrini, Franco Tassi, Juan Diego Rodriguez‐Blanco

**Affiliations:** ^1^ iCRAG Department of Geology School of Natural Sciences Trinity College Dublin Dublin Ireland; ^2^ Department of Geology School of Natural Sciences Trinity College Dublin Dublin Ireland; ^3^ CNR—Consiglio Nazionale delle Ricerche Istituto di Geoscienze Florence Italy; ^4^ Dipartimento di Scienze della Terra – Università di Firenze Florence Italy

**Keywords:** basalt, carbonation, CCS, CO_2_, crystals, glass, reduction

## Abstract

Carbon capture and storage (CCS) technology aims to decrease atmospheric carbon dioxide (CO_2_) levels. Subsurface mineral carbonation is the safest method of CCS, where water‐dissolved CO_2_ is injected into the subsurface and reacts with mafic or ultramafic minerals that release cations (Ca^2+^, Mg^2+^, Fe^2+^), resulting in the formation of carbonate minerals. However, more research is needed to effectively implement CCS in basaltic reservoirs. In these experiments, it is shown how two different samples (basaltic glass and basaltic crystals) react to carbonation under identical conditions: a pH of around 6.5, reaction times of 2 and 5 days, temperatures of 100°C and 200°C, and absolute pressures from 70 to 85 bar(a) at the operating temperatures. The results show partial dissolution of mineral phases, formation of new alteration phases, and carbonate precipitation. This study aims to identify key variables to analyze in such experiments to evaluate the formation of CO and CH_4_ as an alternative reaction pathway during mineral carbonation of injected CO_2_ in basalts. Ultimately, this research aims to lay the groundwork for future studies on basalt carbonation and on how the CCS process might be influenced by CO_2_ reduction to CO and CH_4_.

## Graphical abstract

Short‐term CO_2_–water reactions show that crystalline basalt undergoes more extensive carbonation than basaltic glass and is associated with detectable H_2_ and higher CO and CH_4_ concentrations. This contrast is consistent with the greater early reactivity of Fe‐ and Mg‐bearing crystalline phases, indicating that basalt mineralogy influences coupled mineral carbonation and redox pathways during geological CO_2_ storage.

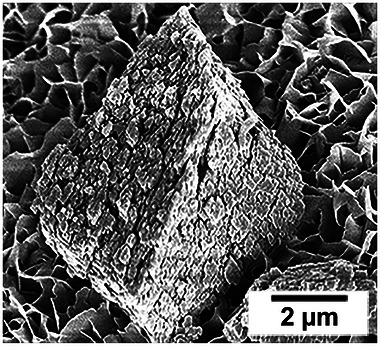

## 1 Introduction

Over the past century, the intensification of the greenhouse effect in Earth's atmosphere has been observed, leading to significant environmental changes, including a projected increase of global surface temperatures of 1.4°C to 5.9°C between 1990 and 2100, an accelerated rate of temperature rise, rising sea levels, the melting of glaciers, and both warming and gradual acidification of the oceans. Most of the carbon dioxide emissions in the atmosphere are attributed to fossil fuel combustion and land‐use changes [1–3], and the result of these human activities contribute to altering the carbon cycle, releasing approximately 42 billion tons (gigatons) of CO_2_ to the atmosphere each year. To reduce emissions and their side effects, new ideas and technologies have been proposed to address this problem. Among them, carbon capture and storage (CCS) via mineral carbonation is the most promising solution. CCS is a crucial technology for achieving long‐term climate targets, as it directly reduces CO_2_ concentrations at emission sources. Without the implementation of CCS, the financial cost of limiting CO_2_‐equivalent concentrations to 450 ppm by 2100 could increase by as much as 138% compared to scenarios incorporating CCS [4].

One of the most promising methods for CCS is carbon mineralization, consisting of chemical reactions involving CO_2_, dissolved as HCO_3_ in pore water, to form mineral phases within rocks. These processes effectively lock away carbon on a geological timescale, proceeding as follows: (1) due to mineral replacement reactions, the formation of reaction edges around individual grains takes place, (2) the precipitation of new mineral phases occurs under low flow conditions, and (3), the pre‐existing mineral phases are altered to clay minerals and subsequently dissolved. Overall, mineral‐water interaction processes result in the formation of new carbonate minerals such as calcite (CaCO_3_), aragonite (CaCO_3_), dolomite (CaMg(CO_3_)_2_), magnesite (MgCO_3_), siderite (FeCO_3_), and dawsonite NaAl(CO_3_)(OH)_2_. Calcite, aragonite, and dolomite are the most important and widespread carbonate minerals at normal surface conditions [5]. Siderite and magnesite, which are isostructural with calcite, are carbonates found particularly in hydrothermal systems [6]. The Earth's crust conditions favor a solid solution between calcite, magnesite, and siderite [7], including other divalent cations (e.g., Sr^2+^, Ba^2+^, Pb^2+^, etc.). Chemical reactions depend upon the pore water chemistry, rock mineralogy, and the length of the migration path.

The rate‐limiting step in this reaction is believed to be the release of divalent cations [8, 9]. Consequently, any process that slows basalt dissolution would impede carbon storage efforts. In recent years, this mechanism has been investigated in other types of rocks, including mafic and ultramafic rocks, to better understand its implications for carbon storage [9–17]. During the alteration of mafic and ultramafic rocks, there is release of divalent ions such as Fe^2+^, and their reaction with fluids and oxidation promotes the reduction of CO_2_ into CO and CH_4_. Therefore, gas concentrations in hydrothermal fluids can be influenced by various reactions between fluids and minerals that are in a state of metastable equilibrium, as well as by gas contributions from host rock dissolution and/or magma degassing [18–24].

This study aims to investigate the early stages of CO_2_‐water‐basalt interactions under high‐temperature and high‐pressure conditions that approach the supercritical state of CO_2_, to increase CO_2_ reactivity during the experiments, and to verify whether the formation of more reactive phases occurs in short‐term experiments (2 and 5 days). This information was used to evaluate the potential production of CO, CH_4_ and H_2_ besides the carbon mineralization of CO_2_, and their rate in early‐stage reactions.

## 2 Results

### 2.1 Mineral Characterization

**FIGURE 1:**
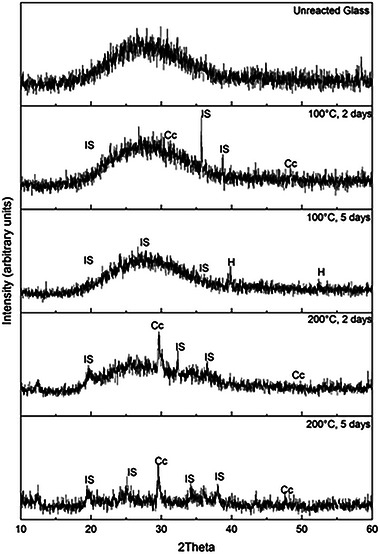
XRD patterns for basaltic glass samples and for each experiment (Cc = calcite, IS = Ilmenite‐smectite, H = hematite).

**FIGURE 2:**
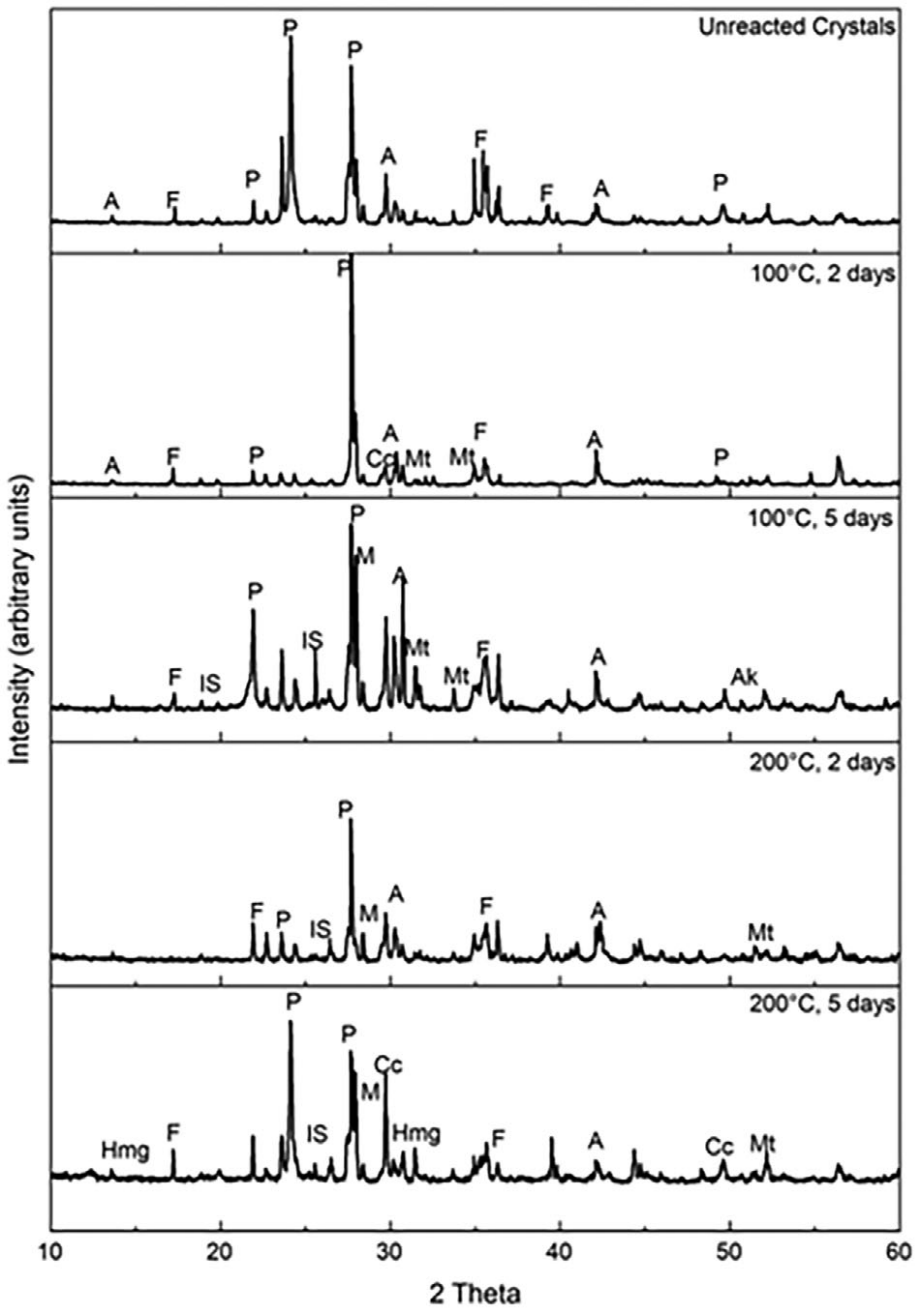
XRD patterns for basaltic crystals samples and for each experiment (P = Plagioclase, F = Forsterite, A = Augite, Cc = calcite, IS = Ilmenite‐smectite, H = hematite, M = Magnesite, HY = Hydromagnesite, Mt = Magnetite, Ak = Ankerite).

Inspection of the XRD patterns (Figures 1 and 2) indicates that variations in the experimental conditions resulted in significant changes in the mineralogical phase assemblages.

Carbonation experiments performed on basaltic glass (Experiments 1–4) reveal a reaction pathway dominated by limited dissolution and secondary phase precipitation, with the glass remaining the dominant component under all investigated conditions. At 55 bar (a) and temperatures of 100–200°C, the products consisted of 97–99% residual glass, indicating only minor reaction progress (Figure 1). Carbonate formation was incipient, with Mg‐calcite appearing in all reacted samples and siderite forming at higher temperature (200°C), suggesting increasing Fe mobility under more thermally enhanced conditions. With increasing temperature and reaction progress (notably in Experiment 3), the proportion of crystalline products increased modestly (up to ∼11%), with clay minerals becoming the dominant secondary phase relative to carbonates. In contrast, higher pressure at lower temperature (Experiment 4; 59 bar (a), 100°C) resulted in negligible reaction, with only trace clay formation and no significant carbonate precipitation. These observations indicate that basaltic glass undergoes relatively slow carbonation, primarily through incongruent dissolution followed by precipitation of Mg‐bearing carbonates and clay minerals, with reaction progress strongly enhanced by temperature rather than pressure.

In contrast, experiments conducted on crystalline basaltic assemblages (Experiment 5–8), initially composed of forsterite, augite, and plagioclase, exhibit a markedly different and more advanced reaction pathway characterized by mineral replacement and compositional redistribution among primary silicates (Figure 2). Across all conditions, the system remains fully crystalline, but significant changes in modal mineralogy occur, including variations in the relative abundances of augite and plagioclase and, in some cases, an apparent enrichment in forsterite, particularly at higher temperature (200°C). Carbonation is consistently expressed through the formation of Mg‐carbonates, primarily magnesite (1%–2%), with hydromagnesite observed in one experiment, indicating locally evolved fluid compositions and possibly higher water activity or CO_2_ supersaturation. These results suggest that carbonation proceeds via direct reaction of Mg‐bearing silicates, especially olivine, leading to the formation of stable carbonate phases while preserving a largely crystalline framework. Compared to glass‐bearing systems, crystalline starting materials promote more efficient and mineralogically controlled carbonation, with limited clay formation and a stronger coupling between silicate transformation and carbonate precipitation. Phase analysis of the experimental products (Table 1) further indicates that, in addition to carbonate phases, iron oxides (magnetite) and clay minerals were also formed, with their relative abundances varying as a function of the experimental conditions; some samples contain mixed clay assemblages likely comprising kaolinite and illite‐smectite.

**TABLE 1:** Mineral percentage detected in original samples and after each experiment.

Sample	Condition	Experiment	Mineral percentage
Basaltic Glass	Unreacted Sample	—	100% glass
Basaltic Glass	100°C, 2 days	Experiment 1	99% glass; 1%Mg‐Calcite
Basaltic Glass	100°C, 5 days	Experiment 4	99% Glass; 1% clays; <1% Hematite
Basaltic Glass	200°C, 2 days	Experiment 3	89% glass; 11% crystals (26% Mg‐Calcite; 74% clays)
Basaltic Glass	200°C, 5 days	Experiment 2	97% glass; 3% crystals (46% Mg‐Calcite; 44% Siderite;10% clays)
Basaltic Crystals	Unreacted Sample	—	7% Forsterite; 14% Augite; 79% Plagioclase
Basaltic Crystals	100°C, 2 days	Experiment 8	1% Forsterite; 59% Augite; 38% Plagioclase; 2% Magnesite;<1% Magnetite
Basaltic Crystals	100°C, 5 days	Experiment 5	1% Forsterite; 63% Augite; 33% Plagioclase; 3% Magnesite; <1% Magnetite; <1% Clays;<1% Dolomite; <1% Ankerite
Basaltic Crystals	200°C, 2 days	Experiment 7	12% Forsterite; 42% Augite; 44% Plagioclase; 2% Magnesite; <1% Magnetite; <1% Clays
Basaltic Crystals	200°C, 5 days	Experiment 6	10% Forsterite; 21% Augite; 59% Plagioclase; 2% Hydromagnesite; 8% Magnesite; <1% Clays;<1% Calcite; <1% Hydromagnesite;<1% Magnetite

In the basaltic glass experiments (Figure 1), limited formation of new phases was detected at 100°C after 2 days of reaction, whereas phase alteration and formation of new crystalline solids became markedly more pronounced in the 200°C experiment after 5 days. In contrast, carbonation experiments conducted on basaltic crystals (Figure 2) exhibited substantial alteration and formation of secondary phases even under the 2‐day, 100°C conditions, with considerably enhanced alteration observed after 5 days at 200°C. Quantitative phase analysis of the experimental products, performed using TOPAS (Table 1), indicates that, in addition to carbonate phases, iron oxides (magnetite) and clay minerals were also formed, with their relative abundances varying as a function of the experimental conditions. Some samples contain a mixture of clay minerals, likely comprising kaolinite and illite–smectite.

Results from Experiments 1 to 4 indicated that carbonation at 100°C was very limited at the tested conditions. Extending the reaction duration did not significantly impact the formation of new carbonate phases. At 200°C, only a few newly formed phases were observed after 2 days, but this number increased over time. The major alteration phases identified by X‐ray diffraction (XRD) in these experiments include clays and calcite, whereas hematite was also detected in minor quantities (<1%). In the experiments with crystals (Experiments 5 to 8), the original phases (forsterite, augite, and labradorite) were abundant (>90%, Table 1), as the experimental conditions did not allow the complete dissolution of these minerals in short‐time dissolution‐precipitation experiments. In the experiments conducted at 100°C, the main alteration phases formed after 2 days include calcite and magnetite. After 5 days, the alteration phases included magnesite, and traces of magnetite, and clays (montmorillonite‐illite). In the experiments carried out at 200°C, after 2 days of reaction, magnesite, montmorillonite‐illite, and magnetite were found, and after increasing the time of reaction to 5 days, hydromagnesite was also identified in addition to the phases formed at 2 days. The experiments showed that the weight % of carbonate minerals originating from the glass did not exceed 2% in the experiments performed at 100°C. The main carbonate phase identified was magnesite (Figure 2). Furthermore, the significant newly formed phases observed include a mixture of clay minerals, likely including kaolinite and illite–smectite (Figure 1), chlorite, and hematite.

**FIGURE 3:**
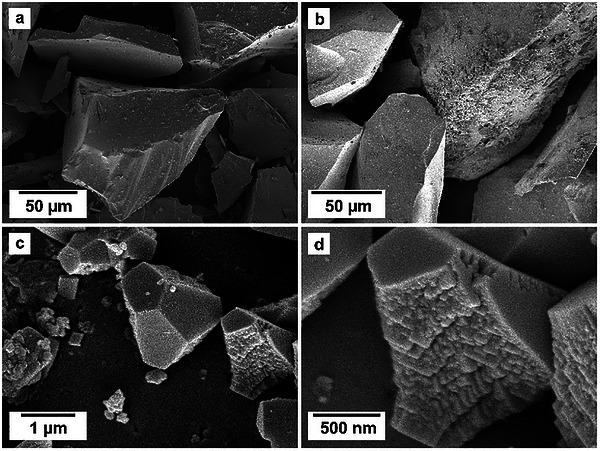
SEM photomicrographs of solids obtained at 100°C in the experiments with glass showing the occurrence of the onset of alteration of basaltic glass samples (a,b) and partially‐developed calcite‐type crystals formed following carbonation of the sample (c,d).

**FIGURE 4:**
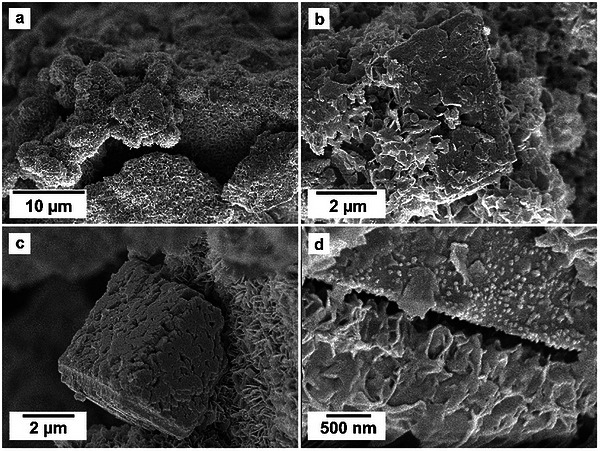
SEM photomicrographs of solids obtained at 200°C in the experiments with glass showing the occurrence of calcite‐type crystals formed on the surface of the original sample (b, c) and presence of diffuse alteration on the surface of the original sample (a–c) showing detachment from the surface of the host (d).

**FIGURE 5:**
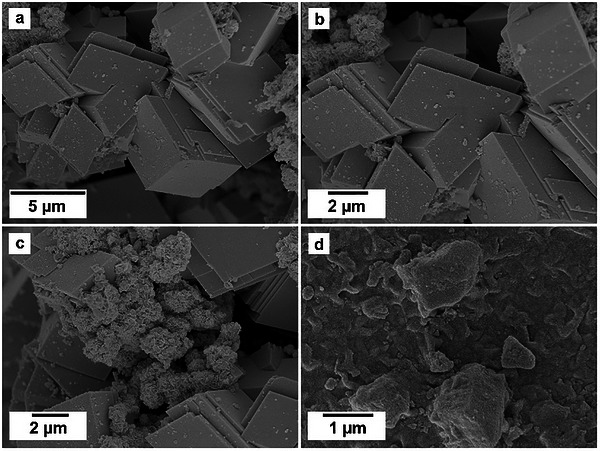
SEM photomicrographs of solids obtained at 100°C in the experiments with crystals showing the occurrence of rhombohedral (calcite‐type) crystals formed (mainly magnesite) on the surface of the original sample (a–c) and presence of alteration of the surface of the host (c, d).

**FIGURE 6:**
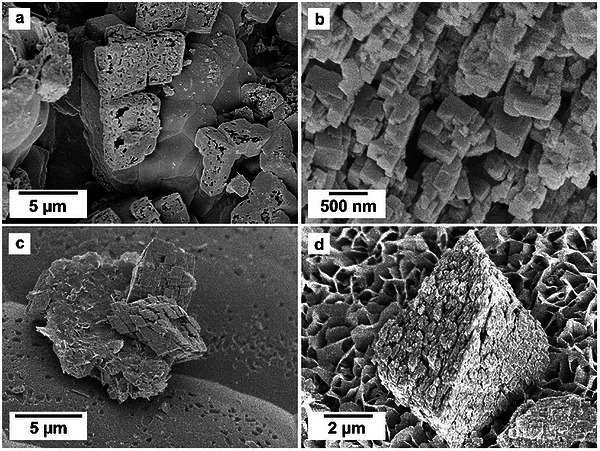
SEM photomicrographs of solids obtained at 200°C in the experiments with crystals showing the occurrence of rhombohedral (calcite‐type) crystals (mainly magnesite) with formed on the surface of the host rock (a–d) and presence of hydromagnesite (flakes) (d). Many of the magnesite crystals show pitted surfaces with high density of defects and mesocrystal‐like morphologies.

The formation of alteration phases on the basaltic glass and crystals surfaces was observed by SEM imaging (Figures 3, 4, 5, 6). In the experiments with basaltic glass, Experiment 1 (conducted at 100°C for 2 days) showed newly formed crystals smaller than 5 µm (Figure 3). However, in Experiment 4 (100°C for 5 days), rhombohedral crystals of magnesite with sizes approximately 5 µm were observed (Figure 3). In the experiments with basaltic glass at 200°C (Experiments 2 and 4, Figure 4), carbonates were found on the surface of the glass after 2 days of reaction. After 5 days, the glass was covered by an alteration layer. In contrast, in the experiments with basaltic crystals (Experiment 8; 100°C for 2 days, Figure 5) showed rhombohedral carbonate crystals larger than 5 µm. Increasing the reaction time to 5 days (Experiment 5, Figure 5) revealed new phases, such as magnesite, ankerite, magnetite and illite‐montmorillonite. Finally, in the experiments conducted at 200°C (Figure 6), carbonation‐formed crystals remained larger than 5 µm after 2 days (Experiment 7); however, after 5 days of reaction (Experiment 6), calcite became more pervasive and covered the entire surface of the original crystals. In experiments using crystalline starting materials, carbonate phases constituted approximately 1 wt.% of the solid products after 2 days at 100°C, increasing to 3 wt.% after 5 days. In these experiments, surfaces showed incipient to pervasive alteration expressed as progressive roughening and diffuse disruption of the substrate, locally leading to detachment. Calcite‐type crystals were sparse to moderately abundant and occurred directly on altered surfaces, ranging from small, poorly defined forms to more distinct but variably developed morphologies.

In contrast, substantially higher degrees of carbonation were achieved at 200°C, with carbonate contents reaching 2 wt.% after 2 days and increasing further to ∼10 wt.% after 5 days. The primary carbonate phases identified in the crystal experiments were magnesite, although calcite and hydromagnesite were also detected in 200°C and 5 days experiment, while dolomite and ankerite were detected in 100°C and 5 days experiment; however, these other carbonates were not quantifiable using TOPAS (Table 1). Additionally, other alteration phases have been observed, including montmorillonite‐illite (Figures 1 and 2), iron oxides such as magnetite (Figure 2). In these experiments, well‐defined calcite‐type rhombohedral crystals were consistently developed on the host surface. These crystals varied in abundance and degree of development, and commonly exhibit pitted surfaces, a high density of defects, and mesocrystal‐like textures. They were locally associated with platy‐to‐flake‐like aggregates of hydromagnesite, forming composite surface assemblages.

In the experiments using crystalline basaltic starting materials (Figures 5 and 6), SEM observations indicate more extensive surface alteration and carbonate precipitation than in the glass runs, consistent with the fully crystalline products identified by TOPAS. At 100°C, rhombohedral carbonate crystals larger than 5 µm on the crystal surfaces were found. At 200°C and 2 days (Experiment 7), progressive development of surface alteration products occurred, and after 5 days at the same temperature, Experiment 6 exhibited more pervasive secondary phase coverage, including hydromagnesite in addition to magnesite. Based on the quantitative phase analysis, the carbonate phases formed in the crystalline experiments are best assigned primarily to magnesite. The modal data further indicates that total carbonate abundances in the crystal experiments remained low overall, generally on the order of ∼1‐2 wt.%. In addition to carbonate phases, other alteration products were also found, including iron oxides such as magnetite, clay minerals (likely comprising kaolinite and illite‐smectite) and amorphous silica.

### 2.2 Gas Analyses

The data on the post‐experiment gas compositions are presented in Table 2, where the percentages of CO_2_ after the experiments are included, as well as those of CH_4_, CO, and H_2_.

**TABLE 2:** List of Experiments and relative gas compositions.

Parameters	Experiment 1	Experiment 2	Experiment 3	Experiment 4	Experiment 5	Experiment 6	Experiment 7	Experiment 8
sample	Glass	Glass	Glass	Glass	Crystals	Crystals	Crystals	Crystals
weight (g)^a^	1.8044	1.8023	1.8024	1.803	1.8048	1.8008	1.8001	1.8
Water/rock ratio	5.542008424	5.548465849	5.548158012	5.546311703	5.540780142	5.553087517	5.555246931	5.555555556
time (days)	2	5	2	5	5	5	2	2
Charging pressure at 21°C (bar (a))^b^	55	55	55	59	55	55	59	53
Operating pressure at start experiment (bar(a))	70	83	82	71	70	85	81	71
Operating pressure at end experiment (bar(a))	49	57	62	50	64	70	70	74
Post‐cooling and sampling pressure (bar(a))	33	39	42	33	44	58	58	56
T (°C)	100	200	200	100	100	200	200	100
CO_2_ (vol%) ^c^	92.3	98.4	98.9	93.8	98.9	98.6	98.8	98.9
N_2_ (vol%)	5.92	1.25	0.91	5.37	0.87	1.12	0.98	0.87
O_2_ (vol%)	1.61	0.21	0.15	0.84	0.11	0.08	0.12	0.09
Ar (vol%)	0.15	0.03	0.01	0.12	0.01	0.02	0.02	0.02
CH_4_ (vol%)	b.d.l.^d^	0.054	0.0018	b.d.l.	0.081	0.095	0.066	0.072
H_2_ (vol%)	b.d.l.	b.d.l.	b.d.l.	b.d.l.	0.008	0.021	0.013	0.014
CO (vol%)	0.0047	0.0098	0.011	0.0064	0.013	0.015	0.011	0.016

^a^ Grams.

^b^ Absolute pressure.

^c^ Volume percentage.

^d^ Below detection limit.

In the glass experiments, the main gas detected was CO_2_, with concentrations ranging from 92.3% to 98.9%. The levels of CO were significantly lower, falling between 0.0047% and 0.011%. CH_4_ was also present in small amounts, measured at 0.0018% in experiments conducted at 100°C after 5 days. However, a higher concentration of 0.054% was observed in the 200°C experiment after the same duration. Notably, molecular H_2_ was never detected in any of the glass experiments, as its levels did not reach the instrument's detection limit.

In contrast, experiments conducted using basaltic crystals as samples revealed that both temperature and duration had a significant impact on CO_2_ concentration. A CO_2_ level of 98.6% was recorded in a 5‐day experiment conducted at 200°C. Among the other carbon phases analyzed, CH_4_ showed higher concentrations, peaking at 0.095% in Experiment 6, while CO remained constant at 0.01%. Unlike the previous glass experiments, H_2_ showed different percentages in the crystal experiments, ranging from 0.008% to 0.021%. Additionally, in both the glass and crystal experiments, N_2_, O_2_, and Ar were attributed to residual air in the reactor or sampling system, together with N_2_ introduced during water de‐aeration.

## 3 Discussion

### 3.1 Carbonation in Basaltic Glass and Crystals

The carbonation of basaltic rocks involves complex interactions between host mineral dissolution, ion release, and carbonate precipitation, processes that are strongly influenced by experimental conditions such as temperature, pressure, solution composition, and redox state. When approaching conditions near the supercritical state of CO_2_, elevated pressures increase CO_2_ solubility in aqueous solutions and inhibit the subsequent transformation of the primary minerals into clay minerals [25]. Conversely, CO_2_ solubility decreases with increasing temperature and salinity, thus environments with hot, highly mineralized formation waters may limit CO_2_ dissolution and, consequently, carbonation [26]. In our experiments, by taking advantage of the increase in volume of the CO_2_ due to the increase in heat, we manage to reach pressures up to 85 bar(a) at 200°C, with CO_2_ nearly reaching supercritical state, which acts as a dense, high‐diffusivity fluid, and favors carbonation reactions, as it applies in Carbfix [27].

Different basaltic phases exhibit widely varying dissolution kinetics [28]. Forsterite is typically the most reactive mineral and is rapidly altered during carbonation [29], whereas other phases, including enstatite [30], anorthite [31], and basaltic glass, dissolve more slowly, releasing Ca^2+^, Mg^2+^ and Fe^2+^ [32]. As observed in previous experimental studies on basalt carbonation, the first ions released are Mg^2+^ and Fe^2+^, because of the rapid dissolution of olivine and pyroxene even at low temperatures within the range of 5 to 75°C [33, 34]. The release of Ca^2+^ is a consequence of the dissolution of plagioclase but occurs at a slower rate compared to olivine under the same conditions, as previously stated [33]. Regarding the dissolution of basaltic glass, the first elements released were Ca^2+^, Mg^2+^, Na^+^ and K^+^, within a range of temperature of 25°C to 90°C [35–37].

In our experiments (Table 1), basaltic glass showed minimal alteration over short timescales (2 days), with the formation of secondary phases occurring only after prolonged reaction times (5 days) and elevated (200°C) temperatures. In contrast, experiments using crystalline basalt experienced more complex dissolution kinetics due to the presence of multiple phases (forsterite, augite, and labradorite). Forsterite releases Mg^2+^ preferentially, while labradorite releases Ca^2+^, and Fe^2+^ originates primarily from impurities in forsterite and augite. All these cations are essential for subsequent carbonate formation. In contrast, plagioclase exhibits lower reactivity compared to other Ca‐ and Mg‐rich silicates [38], promoting the formation of Al‐bearing secondary phases (proto‐Al‐hydroxides, gibbsite, clays) which can limit the number of sites available for carbonate crystallization [39].

During this basalt‐water‐CO_2(aq)_ interaction process, carbonate formation competes with the precipitation of other secondary phases, such as clays and zeolites [40]. The extent and nature of carbonation in basaltic rocks result from a complex interplay of mineralogy, solution chemistry, temperature, pressure, and redox evolution. Several studies [41–43] have examined how cation activity influences the precipitation kinetics of carbonates. Forsterite is widely regarded as the most reactive basaltic mineral during carbonation reactions and favors the formation of Mg‐carbonates such as magnesite [44, 45]. Besides, basalt carbonation experiments conducted at 100°C under elevated pressure have been reported to favor calcite precipitation [14]. Experimental studies further show that magnesite can precipitate rapidly at temperatures of 90–120°C and a pressure of around 100 bar (a) [41–43]. Under these conditions, the kinetic barriers that normally inhibit magnesite formation at lower temperatures, particularly the strong hydration shell surrounding Mg^2^^+^ ions [46], can be overcome, whereas at lower temperatures hydrated Mg‐carbonates such as nesquehonite tend to precipitate instead [42]. This aligns with previous study [47], which observed only ferroan magnesite during tholeiitic basalt carbonation at 50–200°C and pCO_2_ = 300 bar. These experiments consistently produced magnesite and amorphous silica as dominant secondary phases. The release of Ca^2+^ ions from minerals such as labradorite or other feldspars could potentially be translated into the crystallization of calcite [48], aragonite [49] or dolomite (in the presence of Mg^2+^ ions) [50]. Calcite is more soluble than dolomite across a wide range of conditions [51], particularly at temperatures below ∼100°C in the presence of CO_2_ [52–54]. This could lead to an “underestimation” of the calcite content in the experiments performed in this work at 100°C compared to the corresponding experiments at 200°C. In contrast, the behavior of Fe is more complex due to its nature. Siderite may act as a buffer and dissolve in less than 6 h at 7.5 pH, whereas changes in redox potential toward oxidizing conditions would favor Fe^3+^‐bearing oxides or hydroxides such as hematite, magnetite, or goethite [55]. Redox conditions, influenced by the Fe content and mineralogy of basalt, play a critical role in carbonation reactions. Initial oxygen‐rich conditions lead to precipitation of Fe^3^^+^‐bearing phases, such as iron oxides (e.g., hematite) and oxyhydroxides (e.g., goethite), which can become incorporated into silica‐rich interfacial layers, slowing olivine dissolution. As O_2_ is gradually consumed in closed systems, Fe^3+^ phases tend to destabilize, favoring the formation of more soluble Fe^2+^‐bearing minerals [56, 57]. Augite, with the highest Fe content among the minerals studied, releases Fe during dissolution, which predominantly precipitates as low‐solubility iron oxides, inhibiting siderite formation under experimental conditions [58, 59]. As a result of this process, it is possible to observe an oxidation of Fe that lead to precipitation of iron oxides such as magnetite, and the formation of the latter plays a further role in other reactions such as the production of molecular hydrogen which was detected after the carbonation experiments of basaltic crystals but not for basaltic glass, further highlighting how in the latter there was not sufficient release of Fe from the sample during the experiments.

The morphology and surface coverage by precipitated carbonates are time‐dependent; prolonged experiments produce euhedral carbonate crystals that may tend to cover host mineral surfaces (Figures 3, 4, 5, 6). However, previous work on basaltic glass from Stapafell [60] showed that the formation of calcite did not inhibit basalt dissolution. Carbonate precipitates in this work experiments formed discrete prismatic crystals rather than continuous passive coatings, in way that suppression of mineral dissolution requires structural similarity between the dissolving and precipitating phases [61]. Therefore, carbonation of basaltic glass proceeds without significant kinetic inhibition by carbonate coatings.

The experiments show that carbonation in these short‐time experiments is more extensive in crystalline basalts, where forsterite and augite react more readily, while labradorite appears to be only partially altered. This greater reactivity is reflected in the more abundant formation of magnesite, together with secondary phases such as illite–smectite clay minerals. In contrast, carbonation of basaltic glass was slower and was characterized mainly by the formation of calcite, accompanied by clay minerals. From a CCS perspective, these results indicate that the relative proportions of crystalline phases and glass in mafic rocks such as basalts can strongly influence carbonation efficiency and, therefore, the overall mineral trapping potential of the system [66].

### 3.2 Reduction of CO_2_ to CO and CH_4_

Gas analyses conducted at the end of the experiments indicate that, in addition to mineral carbonation, secondary reactions involving CO_2_ occurred under the applied hydrothermal conditions. The formation of CO and CH_4_ (Table 2) varied as a function of experimental duration and starting material, suggesting that both reaction kinetics and mineralogical controls influence the extent of CO_2_ reduction during carbonation. Overall, experiments conducted with basaltic crystals produced higher concentrations of CO and initial CH_4_ formation than those using basaltic glass, implying that crystalline phases may promote redox reactions more effectively than amorphous materials. CH_4_ was detected only in longer‐duration experiments involving basaltic glass, whereas in basaltic crystal experiments it was already present after shorter reaction times. This contrast likely reflects differences in surface reactivity, Fe availability, and fluid–rock interaction pathways between the two starting materials.

The occurrence of CO and CH_4_ is consistent with CO_2_ reduction via Fischer–Tropsch–type reactions, which have been documented in hydrothermal systems containing Fe‐bearing minerals [62,63]. Previous studies have demonstrated that the release of Fe^2+^ into solution can catalyze CO_2_ reduction even at temperatures lower than those employed here [64], supporting the interpretation that Fe mobilization during basalt alteration played a key role in facilitating these reactions. However, the efficiency and extent of CO_2_‐to‐CH_4_ conversion remain highly uncertain. While serpentinization experiments reported relatively high CH_4_ yields during olivine [62], subsequent work [65] under comparable conditions yielded conversion rates that were an order of magnitude lower, leading to the conclusion that earlier results were likely influenced by thermogenic contamination. These discrepancies highlight that CH_4_ formation under hydrothermal conditions is strongly dependent on experimental setup and that even small amounts of CH_4_ detected in the present experiments should be interpreted cautiously.

From the perspective of mineral carbonation, neither CO nor CH_4_ contributes positively to CO_2_ storage. CO is largely inert with respect to basaltic minerals and does not participate in carbonate formation. Nevertheless, its internal generation represents a sink for CO_2_ that reduces the amount available for permanent mineralization. CH_4_ does not contribute to silicate dissolution or carbonate precipitation [66], although at the trace concentrations measured here, its direct effect on overall carbonation efficiency and fluid properties is likely negligible.

Mineral carbonation relies on the formation of H_2_CO_3_ to drive basalt dissolution and release divalent cations necessary for carbonate mineral formation [67]. CH_4_ is known to affect the density, viscosity, phase behavior, and buoyancy of CO_2_‐rich mixtures when present at appreciable mole fractions [68]. However, the maximum CH_4_ concentration measured here was only 0.095 vol %. At this concentration, the direct effect on bulk thermophysical properties is expected to be negligible; a first‐order ideal‐mixture estimate gives an approximately 0.06% reduction in molar mass and ideal‐gas density. The present experiments, therefore, do not support a reservoir‐scale flow or storage‐capacity effect from the measured CH_4_. Such effects would require accumulation to substantially higher concentrations and should be evaluated using an appropriate equation of state and reservoir‐scale reactive‐transport model. In addition, CH_4_‐bearing fluid mixtures exhibit reduced density and viscosity compared to pure CO_2_, which can alter flow behavior, pressure evolution, and fluid distribution within porous basalt formations [69]. These physical effects may further influence fluid‐rock interaction and carbonate precipitation. Taking together, these observations indicate that such redox reactions involving CO_2_ can influence both carbon mass balance and storage efficiency during experimental basalt carbonation and should be considered when evaluating the performance of hydrothermal carbonation systems.

### 3.3 Generation of H_2_ During Carbonation of Basaltic Minerals

The production of H_2_ during carbonation reactions differed markedly between the two starting materials investigated. Under the experimental conditions applied, H_2_ was detected only in experiments conducted with basaltic crystals, whereas no hydrogen was observed in experiments using basaltic glass. This suggests that mineralogy played a critical role in governing hydrogen generation during basalt carbonation. It remains uncertain whether the absence of H_2_ in glass experiments reflects insufficient reaction time, unfavorable redox conditions, or rapid consumption of hydrogen through secondary reactions, such as methane formation.

H_2_ generation in basalt‐CO_2_‐water systems can be attributed primarily to two reaction pathways: redox reactions involving Fe‐bearing silicate minerals and water‐splitting reactions [70]. These mechanisms differ fundamentally in their mineralogical requirements and reaction products. Mineral redox‐driven H_2_ formation typically involves the alteration of ferrous‐containing minerals such as olivine and pyroxene and is commonly associated with the formation of secondary minerals such as serpentine. In contrast, water splitting is linked to oxide formation and does not require the presence of specific Fe^2+^‐bearing silicates [71, 72].

As CO_2_ dissolves, the resulting decrease in pH promotes the release of Fe^2+^ from minerals such as pyroxene and olivine. These ferrous ions act as electron donors, facilitating the reduction of H^+^ to molecular hydrogen. The detection of H_2_ exclusively in experiments with basaltic crystals therefore suggests that crystalline phases provided more effective sources of reactive Fe^2^^+^ than basaltic glass under the conditions investigated. The potential consumption of hydrogen through subsequent reduction reactions, particularly methanogenesis, must also be considered. If H_2_ was generated transiently in the glass experiments but rapidly consumed during CH_4_ synthesis, it may not have accumulated to detectable levels. This possibility is consistent with the observed occurrence of methane in some experiments and highlights the complex combination between redox reactions and carbon reduction pathways during hydrothermal carbonation. Due to the residual O_2_ concentrations of 0.08–1.61 vol%, the experimental condition couldn't be considered strictly anoxic. Therefore, the O_2_ may have affected the Fe redox pathways by providing an alternative electron acceptor to H^+^ or H_2_O. Consequently, the release of Fe^2+^ from olivine or pyroxene could have been oxidized to Fe^3+^‐bearing oxides, oxyhydroxides, or Fe–Si‐rich interfacial phases without generating an equivalent amount of H_2_ [73]. This would decrease both the recovered H_2_ concentration and the reducing equivalents available for CO_2_ reduction to CO and CH_4_ [74]. Magnetite formation is therefore not a unique proxy for H_2_ generation because it can result from Fe^2+^ oxidation by either water or O_2_ [73]. The effect may have been particularly relevant in glass experiments (1 to 4) which contained 1.61 and 0.84 vol% O_2_, respectively, and produced no detectable H_2_ or CH_4_. However, because O_2_ was measured only after cooling and sampling, and because no oxygen‐free controls were performed, the magnitude of this effect cannot be separated from differences in mineral dissolution, temperature, and reaction duration.

The co‐occurrence of H_2_ generation and CO_2_ mineralization has broader implications for subsurface carbon storage strategies. Mafic and ultramafic rocks offer the potential for a dual‐function decarbonization approach, whereby injected CO_2_ is permanently stored as carbonate minerals while simultaneously driving hydrogen production [75]. Although this coupled process is not yet viable at an industrial scale, the experimental evidence presented here supports the feasibility of such reactions under hydrothermal conditions and underscores their relevance for future research into integrated carbon storage and clean energy generation.

### 3.4 Implication for CCS Processes

Mineral carbonation is a well‐understood, and commercially deployed technique that uses calcium‐ or magnesium‐rich silicates (such as olivine, pyroxene, serpentine), as demonstrated by Carbfix in Iceland [76] and 44.01 in Oman [77]. It is considered one of the safest, most permanent methods of carbon storage, with the added benefit of potentially producing useful byproducts by using industrial waste materials, such as mining tailings [78, 79] and steel slugs [80].

The different reaction speed between basaltic crystals and basaltic glass plays an important role [81]; in fact, it can be seen in this work how, in short reaction times (up to 5 days of reaction), regardless of the temperature, basaltic crystals (and in particular forsterite and augite) tend to have a much faster reaction to CO_2_ than basaltic glass; in early stages of reaction with CO_2_‐charged water, crystalline phases (specifically forsterite and augite) are among the first to dissolve, providing a rapid source of divalent ions (Mg^2+^, Ca^2+^, and Fe^2+^) for carbonate formation. Generally, basaltic glass has higher reactivity than total crystalline basalt over longer periods due to its amorphous, high‐surface‐area nature, and this problem has been seen during long‐time reaction that leads to passivation of the crystals [60]. This means that in the basaltic glass, the reaction can be prolonged and not interrupted due to the formation of new carbonates on the surface of pre‐existing crystals, which inhibit their consumption during carbonation.

In addition to mineralizing CO_2_ in various matrices, a secondary formation of other carbon phases, such as CO and CH_4_, was observed. The presence of CH_4_ and CO as impurities during CO_2_ storage has been studied previously [82, 83], which markedly impacts process efficiency, safety, and physical properties. The primary issue with methane in CO_2_ storage sites is reduced storage efficiency: CH_4_ in the gas mixture or in the reservoir decreases the total CO_2_ storage capacity. Another concern involves permeability in the caprock; CH_4_ can increase the buoyancy of the gas plume, potentially elevating the risk of upward migration or leakage if the caprock's integrity is compromised. CO is an impurity that might already be present in industrial waste gases used for CCS, where the main issues relate to possible adverse effects on transport and injection, which strongly influence the volumetric properties and liquid‐vapor equilibrium of the CO_2_ stream. Nonetheless, the formation of CO is not explicitly listed as a primary, high‐risk factor for CO_2_ storage capacity; the main risks to storage capacity and integrity generally involve pressure buildup (overpressurization), geomechanical risks (e.g., fault reactivation, fractures), and well integrity failure [84].

The presence of H_2_ into CO_2_ storage sites acts as a density reducer, decreasing the overall density of the stored gas [85]. The interaction of hydrogen and water results in minimal dissolution and precipitation reactions with basalt, preserving the integrity of rock structures [86]. Furthermore, it has been suggested that generating hydrogen during CO_2_ storage in mafic and ultramafic bodies could optimize decarbonization efforts [75]. This method, termed “orange hydrogen,” not only aids in CCS but also produces a valuable clean energy byproduct, enhancing economic viability.

## 4 Conclusions

Short‐term hydrothermal experiments indicate that basaltic crystalline materials undergo more extensive carbonation than basaltic glass under the conditions investigated. This result contrasts with some previous studies emphasizing the enhanced reactivity of basaltic glass due to its resistance to passivating layer formation. The present findings indicate that elevated temperatures and pressure conditions strongly accelerate carbonation kinetics and favor more rapid mineral‐fluid reactions in crystalline basalts. The lower recovered calcite abundance in the 100°C experiments could reflect differences in Ca release, carbonate nucleation, precipitation kinetics, and/or post‐precipitation dissolution. However, no post‐reaction fluid analyses of Ca, alkalinity, or dissolved inorganic carbon were performed, and saturation indices could therefore not be calculated. Differential calcite dissolution should consequently be regarded as a possible, but unverified, explanation.

A closed Fe and electron balance could not be established from the available measurements. The Rietveld results represent relative phase abundances; magnetite was below 1 wt%, and no measurements of dissolved Fe or phase‐specific changes in Fe oxidation state were made. Likewise, the GC results provide gas‐phase mole fractions but not totally evolved gas inventories. Consequently, the association between crystalline Fe‐bearing phases, magnetite, H_2_, CO, and CH_4_ is interpreted as consistent with an Fe‐mediated redox pathway but does not uniquely demonstrate that pathway or permit apportionment of Fe contributions between olivine and augite. These differences are consistent with greater short‐term availability of reactive Fe^2+^ from Fe‐bearing crystalline phases. However, the absence of aqueous Fe measurements, an absolute solid‐phase balance, and redox‐controlled blank experiments prevents unique attribution of the reduced gases to individual mineral phases.

Hydrogen production followed a similar trend. H_2_ was detected exclusively in experiments involving basaltic crystals and was absent from glass carbonation experiments. Hydrogen generation likely resulted from redox reactions and water–rock interactions facilitated by Fe‐bearing minerals, rather than from serpentinization processes, which were not favored under the experimental conditions applied.

Overall, the results indicate that, under the conditions explored in this study, basaltic crystalline materials are more effective than basaltic glass in promoting carbonation, CO_2_ reduction to CO and CH_4_, and H_2_ generation. From an applied perspective, the concurrent production of CO, CH_4_, and H_2_ during CO_2_ storage in basalt formations may have both beneficial and detrimental implications. While hydrogen generation represents a potential benefit for energy applications, including the production of low‐carbon (“orange”) hydrogen, the presence of gases such as CO and CH_4_ may reduce effective pore space and limit CO_2_ storage efficiency. The combination of positive aspects (rapid mineralization of CO_2_ and H_2_ generation) and negative aspects (pore space reduction and trapping hysteresis) highlights the importance of considering coupled geochemical and physical processes when evaluating basalt formations for long‐term carbon storage and integrated decarbonization strategies.

## 5 Materials and Methods

### 5.1 Sample Preparation and Description

The crystalline and glass basalts used in this study are from the basaltic dyke on Stapafell Mountain (SW Iceland), that was in‐depth characterized and used in previous carbonation research studies [35, 33, 60, 81], and therefore it serves as a good base sample for correlating our results. The examined dyke did not show any visible signs of alteration. The Stapafell sample was chosen because it has a chemical‐mineralogical composition very similar to the composition of the basalts typical of MORB [87, 88]. The chemical composition of the glass was investigated by previous work [35], which also measured the Fe^2+^/Fe^3+^ ratio, showing that Fe is mostly in the Fe^2+^ form.

In this study, the basaltic glass was crushed using a hammer in plastic bags and sieved to obtain a 45–125 µm size fraction. Fine particles were removed from the glass fraction through a two‐step process. First, gravity sedimentation was used, followed by ultrasonic cleaning of the glass powder with water, followed by cycles using acetone, repeated five times. Once each cycle, the ultrafine suspension was discarded. The resulting glass powder was dried overnight at 60°C. The basaltic crystal samples are the same ones used previously [33] and were prepared as follows: the crystalline basalt was dried at room temperature for several days; Then, it was grounded using a jaw crusher. The finely grained material was subsequently dry‐sieved to obtain the 45–125 µm size fraction. This size fraction underwent gravitational settling to remove ultra‐fine particles and was cleaned ultrasonically five times in deionized water and acetone. Finally, the resulting powder was oven‐dried at 50°C for several days.

### 5.2 Experimental Procedure

The experimental results derived from eight experiments, four performed on basaltic glass and four on basaltic crystals. Two temperatures (100°C and 200°C) were selected, and for each T, two runs of different durations (2 and 5 days) were performed. The experiments took place at the Laboratory of Experimental Petrology and Mineralogy in Florence, using a Parr 5500 micro‐reactor (25 mL capacity). The experimental procedure involved several steps: (1) the sample (approximately 1.8 g) was weighed; (2) it was dried at 60‐degree in an oven to remove moisture; (3) to remove the dissolved air from the Milli‐Q water used for the experiments, N_2_ was flushed inside Milli‐Q water for 2 min before each experimental run; (4) water (10 mL) was added to the vessel together with the solid sample; (5) the vessel containing the experimental charge was sealed and placed in the external heater; (6) CO_2_ was introduced into the still‐cold vessel until the maximum pressure was reached (approximately 55 bar(a)); (7) the temperature was adjusted to the chosen level (either 100 or 200°C) and kept for 2 or 5 days; (8) the temperature was reduced to 21°C, causing a decrease in pressure due to the volumetric contraction of the gas; (9) the gas was sampled in 10 mL vials containing water acidified with carbonic acid, to prevent the solubilization of CO_2_ in the water. Two valves were used, one for the release of the water and the other for the collection of the gas. The sampled gas was promptly analyzed by gas chromatography to prevent data loss on the H_2_, which could escape from the vials if left for a longer time. After the decrease of internal pressure to atmospheric pressure, the vessel was opened, and the solid sample was collected. This wet sample was dried at 60°C in a ventilated oven. This procedure was applied to both the basaltic crystals and basaltic glass runs.

### 5.3 Sample Characterization

#### 5.3.1 Powder X‐ray Diffraction

Powder X‐ray diffraction (XRD) using Cu‐Kα radiation was used on the solid products to detect and quantify the amount of any newly formed crystalline compounds. The XRD analysis was conducted using a Bruker D5000 X‐ray diffractometer, with 0.01° per step from 5 to 70° 2θ at 0.2° min^−1^. Diffract Suite EVA software from Bruker, in combination with the Powder Data File (PDF‐4, The International Centre for Diffraction Data), was used to identify the XRD patterns. Pattern‐matching refinement and quantification of the crystalline phases were performed through Rietveld refinement software (TOPAS) [89]. The Scherrer equation [90] was used to calculate the crystallite sizes from the Bragg peak full‐width half‐maximum (FWHM), and the unit cell parameters were determined with TOPAS [89].

#### 5.3.2 Scanning Electron Microscopy With Energy Dispersive Spectroscopy

The newly formed crystalline phases were characterized using scanning electron microscopy (SEM). Solids were carbon‐coated and placed into a Tescan TIGER MIRA3 FEG‐SEM operating under high‐vacuum conditions and equipped with four Oxford Instruments NanoAnalysis X‐Max 170 mm2 EDS detectors running Oxford Instruments NanoAnalysis AZtecTimed analysis software. To image powders from solution experiments, they were coated with Au‐Pd and imaged with Tescan TIGER MIRA3 FEG‐SEM equipped with two Oxford Instruments X‐Max 150 mm2 EDS detectors running Oxford Instruments AZtec software. SEM analyses were performed using an accelerating voltage of either 5 or 10 kV.

#### 5.3.3 Gas Analysis

Gases produced from the reactions were analyzed for their N_2_, Ar, O_2_, CO_2_, CO, CH_4_, and H_2_ contents using a Shimadzu 15A and a Thermo Focus gas chromatograph equipped with Thermal Conductivity Detector (TCD). The Fluid Geochemistry Lab of the Department of Earth Sciences at the University of Florence supplied gas chromatograph. CH_4_ was separately analyzed using a Shimadzu 14A gas chromatograph equipped with a Flame Ionization Detector (FID). The detection limit for compounds of GC was 0.001 vol% (10 ppmv), and the analytical error of GC was <5%. To collect the gas sample, a needle was inserted into the gas‐out valve of the PARR reactor, and the valve was slowly opened for about 2 s to purge the reactor. Then, the sampling needle was injected into an Exetainer vial sealed with a porous membrane. The vial contained de‐aired Milli‐Q water, and the gas species were analyzed using the gas chromatographic method.

## Conflicts of Interest

The authors declare no conflicts of interest.

## Data Availability

The data that support the findings of this study are available from the corresponding author upon reasonable request.
